# Supplementary material for: Reliable detection and continuous monitoring of memory dysfunction in mild cognitive impairment and healthy aging through adaptive computational phenotyping
Source: PLOS Digit Health. 2026 Sep 15;5(9):e0001686. doi: 10.1371/journal.pdig.0001686 (PMC13577568; doi:10.1371/journal.pdig.0001686)
Supplement: Supplementary file 1 [file pdig.0001686.s007.docx]

# Response to Reviewers

The original paper was reviewed by three expert reviewers, two of which made interesting comments and suggestions and raised significant points. We were impressed by the quality of the comments made, many of which showcase a deep understanding of the subtlest aspects of our work. We believe our revision has addressed all their points in depth. In particular, our revision has focused on:

1. Adjusting several parts of the Results, adding information and additional analyses to our “Reliability and Reliability” section, expanding the statistical model of our “Longitudinal Group Differences” section, and providing a better justification for our conclusions in the “Relationship Between Recall and Recognition Assessments”.
2. Revising the Discussion section to include new sections that detail (a) the relationship of our approach to other digital assessment tools; (b) the relationship between our central parameter (the speed of forgetting) to other parameters within the model and to behavioral data collected during the task; and (c) Other limitations and confounds in our sample and in our data.
3. Revising the Conclusions section to include a new “Future Directions” section, which now clearly outlines the work that remains to be and the possible directions to explore.

We believe that our paper has greatly improved because of these changes, and we want to take this opportunity to thank our Editor and our Reviewers. The following sections describe the detailed changes made in response to every point raised by our Reviewers.

## Reviewer #1

*In this paper, Hake and colleagues test the utility of a newly developed online memory assessment tool (SGMA) using a longitudinal study of 24 patients and 27 controls with mild cognitive impairment. They provide compelling evidence for the utility of SGMA, showing that an 8-minute session is as predictive as a gold-standard clinical test (MoCA). This rigorous work addresses a timely issue with important clinical implications, and I believe it would be of great interest to the readership of PLoS Digital Health.*

1. *The authors show very convincing results for the accuracy of predicting MCI using the SGMA score*
We thank the reviewer for the encouraging comment!

*2. The results in Table 2 suggest that Sex effect (9.77) is comparable in size to the Group effect (-15.33). The sex differences between the MCI and control groups is a major source of concern. While I understand that this precious data is very hard to collect (especially longitudinally), is there any analysis that could be done to alleviate this concern other than including sex as a covariate in the analyses? Moreover, given that single-session SGMA scores already show very high predictive power, wouldn't collecting single session data from a larger cohort be possible? I don't claim that this is required for this work to be published, but it would definitely strengthen it.*

We thank the reviewer for raising this issue. Indeed, the sex imbalance in our sample is perhaps the biggest limitation in our study. We have explicitly acknowledged that in a new “Limitations” section of the revised Discussion. The same section also provides further analysis confirming that sex differences between groups, while being a significant factor in our analysis, does not impact our conclusions. Finally, the possibility of using SGMA in a complementary way (that is, to collect cross-sectional data from a large sample, instead of a longitudinal data from a small sample) is illustrated in a recently published paper (Wilschut et al., 2026, Comm. Medicine) that is now cited in the revised Discussion and Conclusions.

*3. Along the same lines, 13/24 MCI participants exhibited deficits in executive function, language or visual reasoning. How many showed executive function deficits? Should this also be included as a covariate?*
We thank the reviewer for asking this question. Our data show that 13/24 MCI participants showed deficits in domains *other* than long-term memory, but the classification of “amnestic MCI, multiple domains” was made holistically by the visiting neurologists, and does not specify which of the other domains were affected. An analysis of the neuropsychological assessments (see Figure 10) suggests that most patients exhibited lower executive functions than controls (as evidenced by lower scores in the results in the forward and backward span tests and the trail-making tests), but that their scores were not, overall, associated with their SGMA scores.

*4. The authors claim that the use of the computational model parameter (the average of SoF values) yields better results than raw behavioral measurements. To support this claim, the authors should train the classifier on the underlying measures (accuracy and RT) and show reduced predictive power.*
We thank the reviewer for this suggestion. Because of the adaptive nature of our task, a fair comparison between model parameters and behavioral data is unfortunately impossible: model parameters are adjusted based on behavioral responses, and behavioral measures are elicited in response to items that are, in turn, selected on the basis of the underlying parameters. As a result, behavioral measures do not provide any additional information over model parameters. A new section in the revised discussion, titled “Relationship to Behavioral Data”, directly addresses this problem and provides some analysis corroborating our point.

*5. In the RT analysis (Fig 4), the authors show very strong correlations between the measured and predicted RT. However, it's important to disentangle the contribution of the construct of interest and that of no interest, t0. If I understand correctly, t0 is fitted for each participant, but is not directly related to memory decline in any way. Shouldn't the comparison between the measured and predicted RT values after accounting for t0 (and possibly F as well)? Furthermore - while not at the focus of this work - if F, given the collected data, F could be related to executive function, which was found to be lowered in some of the MCI participants.*
We thank the reviewer for this insightful comment. We have addressed these points in the revised “Model Goodness-of-Fit” section of the Results and in the newly added “Relationship to Other Model Parameters” section of the revised Discussion. The revised Results section stresses that *F* and *t*_0_ were fit after the SGMA score was derived. The model fit analysis in Fig. 4 evaluates behavioral predictions that are only based on estimated φ, which therefore absorbs any variance that could also be ascribed to individual differences in *F* or *t*_0_ if they were to be estimated separately. This analysis is intended only as a way to judge whether the model successfully captures our participant’s behavior. Also, while the t_0_ and F parameters varied across individuals, the Speed of Forgetting parameter varied across items, capturing moment-to-moment variability in responses due to the elapsed time and the number of repetitions of each stimulus. Neither t_0_ nor F capture this source of variability across items.

It is also important to notice that, although *t*_0_ and *F* are not directly related to memory function, they are related to cognitive *decline* in general, which often affects visual and motor aspects as well. As the revised Discussion now clarifies, we do find stable group differences in these parameters, which are consistent with published results in the literature. The revised Discussion also acknowledges the limitations of our approach, and suggests possible ways to improve upon our findings.

*6. “The number of items studied during each session varied... depending on their response times and error rates”. Is this a confounding factor? My understanding is that it is not, but it would be useful to clarify that the number of items does not actively affect the inferred SoF during the experiment.*

We thank the reviewer for raising this concern. As the reviewer points out, this is a natural consequence of the adaptive nature of the task, and does not represent a confound *per se*: participants with lower memory function end up memorizing a smaller number of items that are repeated more times. This is now made explicit in the “Study Materials” section of the revised Methods. The different number of items studied does not affect the reliability of our scores, as our answer to the next comment shows.

*7. “The mean SoF values were identified by using the last SoF of each item…” - Could you please show a visualization of the inferred SoF values time course? It would be useful to show that the values indeed converge and stabilize.*

We thank the reviewer for this suggestion. The new plot (showing SGMA rather than SoF) is now added as Supplementary Figure 1.

*8. In addition, have the authors considered the sensitivity of other measures like the SD of the SoF values? This goes back into what we think the underlying source of change in MCI is. Is it an overall increase if SoF? If so, the mean (or median) is a good measure. However, if it is in fact an increase in the frequency of more rare forgetting events, than the SD or IQR might be more sensitive measures.*

We thank the reviewer for this acute observation. Indeed, we have considered this hypothesis and run such an analysis, and found that within-sessions variability in item-specific SGMA values were, on average, higher in healthy controls (mean = 29.91) than in MCIs (mean 22.40). This result is now reported and discussed in the “Relationship Between Recall and Recognition Assessments” section of the revised Results.

*9. When presenting the stability and reliability of the model-based assessment scores, it would be very helpful to present the stability and reliability of the underlying behavioral measures (accuracy and mean RT). In addition, because we know that there is a difference between groups, such a difference could be driving the high correlation value of Pearson r=0.7. This is not necessarily a bad thing, because it is in fact this difference between MCI and controls that the authors are trying to capture. Still, it would be useful to report the reliability using Spearman correlation, which is less sensitive to extreme values. I would even consider reporting them separately for the control and MCI groups (see point 11 below).*
We thank the reviewer for raising these excellent points. The stability and reliability of behavioral measures, and the problems associated with their interpretations, are now discussed and reported in the new section “Relationship to Behavioral Data” of the revised Discussion. The SGMA correlations were lower for MCI (0.56) than for HC (0.65); these results are now reported in the revised “Stability and Reliability of Model-Based Assessment Scores” section of the Results. We found no difference between Spearman and Pearson coefficients, so we continued using Pearson values.

*10. Fig 7A: Some lessons are more discriminative than others between MCI and controls. Can the authors say something about what makes lesson more discriminative? For example, flags, art and constellations are less discriminative. Is that because they are more visual that other lessons? Because there are other strategies people might use to complete these lessons? Understanding this might get even better predictive results.*
We thank the reviewer for this suggestion. The new “Future Directions” session of the Conclusions specifically lists this as one of the most important research avenues to pursue. As a first attempt to examine this question, we have revised the “Longitudinal Group Differences” section of the Results, including the results of an expanded linear model that also includes the stimulus format (visual vs. textual). As the revised manuscript now makes it clear, stimulus format turned out not to have any significant effect, and not to affect the estimates of any other factor.

*11. Fig 7B: As is often the case in such tests, there is still a lot of overlap between the distributions of the two populations. Could the authors please explicitly address this? Is it possible that in the future, a more sensitive measure for predicting the onset of MCI be the *change* in SGMA over sessions? I think this is exactly the point that the authors are trying to make. If this is the case, the reporting the reliability (using correlations of ICC values) in each group separately, and particularly in the control group, is of great importance.*
We thank the reviewer for these suggestions. We have discussed the potential for using changes in SGMA in the new “Future Directions” section of the revised Conclusions. As noted above, the revised Results now report the reliability of patients and controls separately (see our answer to Comment #9, above)

12. *The analysis of the diagnostic accuracy of SGMA is a very strong part of the paper, and a great contribution to the field.*

We thank the reviewer for the encouraging statement!

*13. I think that this analysis should be complemented with a similar analysis that is based on the measured accuracy and RT measures, as the underlying building blocks of SGMA. It might be the case that they are as predictive as SGMA (which is perfectly fine), but knowing it would be very helpful. Please correct me if I'm wrong, because it's possible that the adaptive nature of this task actually makes accuracy and RT less sensitive (as the platform pushed participants toward a common accuracy value, for example, while inferring the SoF. If this is the case, I think that the readers would greatly benefit from a clear explanation of this point.*

We thank the reviewer for this suggestion, which shows a deep understanding of our approach. We have addressed these issues in the new section “Relationship to Behavioral Data” in our revised Discussion. (See our responses to Comments #4 and #9, above).

14. *The authors write that typical assessments that are based on test accuracy confound memory loss and deficits in accessing a memory (e.g., due to executive dysfunction), but that SGMA is potentially immune to this confound. Could you please elaborate on why SGMA is potentially immune to this confound? Furthermore, given that there are patients that exhibited executive dysfunction in this study's cohort, does anything in their SGMA scores or otherwise reflect this dysfunction?*
We thank the reviewer for raising this concern. To address this concern, we have revised the Results section, clarifying our line of reasoning and providing additional evidence to support our belief that SGMA reflects memory maintenance, rather than memory retrieval processes. As described, the evidence is threefold: (1) SGMA scores are correlated across recognition and recall testing modalities; (2) Variability across items is smaller in MCIs than in health controls, while greater frequency of retrieval failures would have resulted in larger variance; and (3) patients with deficits that include other domains (visual, executive functions) show higher SGMA scores, rather than lower.

15. *Given the strong Sex effect, wouldn't it make sense to run the classification analysis with both SGMA and Sex as inputs in a multiple-logistic classifier? Perhaps this is what the authors have already done. Similarly, did the authors consider using both recognition SGMA and recall SGMA in a single prediction model?*
We thank the reviewer for this suggestion. Indeed, we have addressed this and other issues in \the new “Limitations” section of the revised Discussion.

Minor comments
1. *Intro: The authors write “... as they lacked reliability when used in isolation”. Please explain what this means. Is that test-retest reliability? Was the detection accuracy of 90% not reliable?*

We thank the reviewer for spotting this ambiguity; we acknowledge that this was an awkward sentence. We have revised the statement, making it clear that we are referring to the variability in each instrument’s detection accuracy across studies.

2. *Intro: The authors write: “The present study demonstrates, for the first time, the viability of this approach”. Is that really the first time researchers use computational phenotyping to differentiate healthy individuals from diseased ones? Please soften this claim, or make it specific to the test case in question (detection of MCI).*

We thank the reviewer for raising this point. We have revised the sentence limiting its scope to the detection of memory impairments.

3. *Intro: There is a typo in “an individual can hold on to *be* a newly…”*

We thank the reviewer for the careful read of our manuscript! We have fixed the typo.

4. *Intro: “individuals with MCI would exhibit higher SGMA scores” - should be “lower”, right?*
We thank the reviewer for spotting this mistake; the reviewer is correct, and we revised the text to say “lower”.

5. *In “Adaptive Memory Assessment”, the reference “Fig 2” should actually be to “Fig 3”.*

Thank you for noticing that; we have fixed the figure reference.

6. *Currently, Fig 5 is a bit hard to read. It would make more sense to present the 1st to 9th deciles in this plot, and present the full plots (including the 10th decile) in the supplementary.*

We thank the reviewer for this suggestion. We have revised Figure 5 as suggested, and moved the current figure as the new Supplementary Figure 2.

7. *Fig 6: “from panel B” should probably be “from panel A”.*

Thank you for spotting this mistake. The label has been corrected

8. *The longitudinal aspect of this study, with so many repeated measures per participant, is very impressive. The authors have used this to show explicitly that there is minor improvement due to practice (having a small practice effect is an advantage in terms of test-retest reliability), and that this effect is attenuated in MCI. Given the SGMA (like any other single test) still shows a large fraction of false-positive “diagnosis” of MCI in the logistic classifier, it would have been very useful to show the ability of identifying a conversion from healthy control to MCI. This is obviously not at the hands of the authors, however, if they are able to track these participants further into the future, and show that SGMA is able to identify MCI onset, this would be an extremely strong result.*

We sincerely thank the reviewer for this suggestion; we have added this to the “Future Directions” section of the revised Conclusions.

## Reviewer #2:

1*. The authors report results from a mobile cognitive test of memory consolidation and forgetting performed at home on a weekly basis through a year in patients with mild cognitive impairment (MCI) and a control group of healthy age-matched individuals. The model compared test results to group members. The method description, data processing and statistics are sound and descibed well. The introduction and discussion are focused and well balanced. Overall, a significant contribution to the field.*

We thank the reviewer for this assessment!

## Reviewer #3:

*The authors developed the Seattle-Groningen Memory Assessment to address some constraints of conventional neuropsychological tests; specifically their reliance on in-person testing, vulnerability to practice effects, and lack of adaptive features. They conducted a longitudinal study lasting up to one year, recruiting 27 healthy controls and 24 people with amnestic MCI. On a weekly basis, participants engaged in 8-minute memory tasks delivered online. By applying a mathematical model of memory consolidation to trial-by-trial metrics (both response time and accuracy), the team established a "Speed of Forgetting" (SoF) parameter, which was then normalised as the SGMA score. The findings indicated that the SGMA scores were reliable (r = 0.71) and reached a diagnostic accuracy of 87% differentiating between controls and MCI, which is equivalent to the clinician-led MoCA. T tool proved to be stable across various modalities (recall vs recognition) and different sets of study materials.
It’s a rigorous study addressing a genuine issue in clinical practice and with the adjustments below I think it’d be a valuable contribution to the literature.

Major Points:*

1. *My biggest query concerns the tool’s current ability to do the “continuous monitoring” aspect mentioned in the title. While the work successfully differentiated groups (i.e. cross-sectional utility), it’s not yet shown that the tool can track an individual’s specific trajectory of cognitive decline. The abstract asserts that the score is “only barely affected by practice effects”. But Figure 7C and Table 1 indicate a significant positive fixed effect for “Week” (p<0.001). Although the weekly increase is small (0.17 points), it would accumulate to a ~8.8 point gain over a full year. While it’s encouraging that the increase is smaller in people with MCI (i.e. there’s a separation of cases vs controls), I worry that this persistent upward slope creates a confound for a tool designed to track cognitive decline. Describing the tool as less affected than traditional tests would be more precise than claiming it is ‘resistant’, as it’s clearly not immune. Related to this, I'd be interested to know the mean number of repetitions per item for MCI vs control groups e.g. if MCI patients required significantly more repetitions to reach the same level of accuracy within the 8 minutes, it could demonstrate the ‘burden’ of the task perhaps more clearly than just the final SGMA score?*

We thank the review for this observation. As the Revised Results section now makes it clear, the upward slope is still modest—0.2% increase per session. Even with our weekly assessment schedule, this rate is not sufficient to wash away the difference between groups. And, if assessments were made monthly, it would take almost 10 years before the cumulative effects of practice erased the difference between MCIs and controls. Yet, the fact that practice effects persist even in our adaptive, model-based digital assessment suggests some possible avenues for future research, which are now discussed in the “Limitations” section of the Discussion. Because the SGMA test has a fixed duration, the number of repetitions that a participant sees, on the other hand, is determined by the combined effect of two factors: one is their characteristic speed of forgetting and the other is their characteristic response time, which in our model depends on the non-retrieval time *t*_0_. As the new “Relationship to Other Model Parameters” section of the revised Discussion now makes it clear, MCI patients do exhibit both heightened speed of forgetting and and increased non-retrieval time, which results in patients seen less stimuli overall (10.17	+/- 3.29 for MCIs vs. 14.23 +/- 1.67 for controls), but not experiencing a higher number of repetitions (6.14 +/- 1.21 vs. 6.86 +/- 1.56).

2. *I'm impressed by how high engagement seems to have been in this cohort (engaged with weekly testing for 48.5 weeks). Do the authors have any indication why this might be? Is it something unique to this cohort? Or are there lessons from your approach which you think contributed to this which others could learn from? Either way I’d be interested to hear the authors’ perspective on this*.

We thank the reviewer for inviting us to discuss this issue. In fact, we believe that the level of patient involvement we could achieve is one of the benefits of our approach. We have expanded the revised Discussion to include a specific paragraph about the nature and causes of participant involvement in our study.

3. *Related to the above, would this engagement be lower for people with greater cognitive impairment? Would this be an issue for picking up incident dementia i.e. if as people ‘convert’ from MCI to dementia, would a potential inability to engage with the tool impair the ability to detect the ‘conversion’? While the authors mention the necessity of intact sensory/motor function, I think it might also warrant some mention of the potential for psychological distress or frustration felt by patients who perceive their own cognitive decline through repeated testing.*

We thank the reviewer for this comment. To address this point, we have revised the Conclusions to include more information about the level of engagement that our digital assessment tool can afford, and the role that its adaptive nature plays in increasing motivation by allowing participants to obtain a fairly high degree of accuracy, irrespective of the true degree of their impairment.

4. *There isn’t contextualisation of these results between SGMA and established digital platforms Cogstate or Cognitron. SGMA’s primary differentiator seems to be its digital twin modelling of the forgetting curve, whereas other tools frequently rely on raw performance data. The discussion would benefit from a more explicit explanation of why tracking latent parameters through modelling is superior to the raw data tracking used by other systems.*

We agree that a comparison to established digital platforms would be beneficial to the readers of this manuscript. We have added a paragraph in “Relationship to Other Digital Tools” highlighting some of the main differences between SGMA and other digital cognitive assessment instruments (such as CANTAB and Cognitron) as well as newly proposed AI-based diagnostic tools, focusing on the similarities and differences of the different approaches. We have also added a reference to a paper under review at the Journal of Neuropsychology, which we expect to be online soon, in which we compare SGMA with the Amsterdam Cognition Scan, another digital platform (https://osf.io/preprints/psyarxiv/hwsyf).

5. *The abstract has quite a lot of use of subjective intensifiers e.g. "highly diagnostic”, "highly stable", "only barely affected" which risk overstating some of the results, especially for an n=51 pilot study*

We thank the reviewer for this suggestion. We have removed the superlatives from the abstract.

Minor Points
1. *The tool seems to currently focus on memory. Do the authors intend for this to be used as a standalone tool or alongside other tools testing other cognitive domains or planning to add additional modalities cognitive domains?*

We thank the reviewer for this suggestion. We added a list of possible extensions to the new “Future Directions” sections of the revised Conclusions.
