# Supplementary material for: Reliable detection and continuous monitoring of memory dysfunction in mild cognitive impairment and healthy aging through adaptive computational phenotyping
Source: PLOS Digit Health. 2026 Sep 15;5(9):e0001686. doi: 10.1371/journal.pdig.0001686 (PMC13577568; doi:10.1371/journal.pdig.0001686)
Supplement: Peer review history

# Peer review: Decision Letter 0

**DOI:** 10.1371/journal.pdig.0001686.r001

**Authors:** Pengxu Wei, Peter H Charlton

4 May 2026

Response to Reviewers Revised Manuscript with Track Changes Manuscript **Journal Requirements:**

1. Please amend your detailed Financial Disclosure statement. This is published with the article. It must therefore be completed in full sentences and contain the exact wording you wish to be published.

a. State what role the funders took in the study. If the funders had no role in your study, please state: “The funders had no role in study design, data collection and analysis, decision to publish, or preparation of the manuscript.”

b. If any authors received a salary from any of your funders, please state which authors and which funders.

2. Please provide separate figure files in .tif or .eps format.

For more information about figure files please see our guidelines:  LINK

https://journals.plos.org/digitalhealth/s/figures

https://journals.plos.org/digitalhealth/s/figures#loc-file-requirements

3. We have noticed that you have uploaded Supporting Information files, but you have not included a list of legends. Please add a full list of legends for your Supporting Information files after the references list.

**Reviewers' Comments:**

Reviewer #1: In this paper, Hake and colleagues test the utility of a newly developed online memory assessment tool (SGMA) using a longitudinal study of 24 patients and 27 controls with mild cognitive impairment.

They provide compelling evidence for the utility of SGMA, showing that an 8-minute session is as predictive as a gold-standard clinical test (MoCA).

This rigorous work addresses a timely issue with important clinical implications, and I believe it would be of great interest to the readership of PLoS Digital Health.

Major comments

1. The authors show very convincing results for the accuracy of predicting MCI using the SGMA score

2. The results in Table 2 suggest that Sex effect (9.77) is comparable in size to the Group effect (-15.33). The sex differences between the MCI and control groups is a major source of concern. While I understand that this precious data is very hard to collect (especially longitudinally), is there any analysis that could be done to alleviate this concern other than including sex as a covariate in the analyses? Moreover, given that single-session SGMA scores already show very high predictive power, wouldn't collecting single session data from a larger cohort be possible? I don't claim that this is required for this work to be published, but it would definitely strengthen it.

3. Along the same lines, 13/24 MCI participants exhibited deficits in executive function, language or visual reasoning. How many showed executive function deficits? Should this also be included as a covariate?

4. The authors claim that the use of the computational model parameter (the average of SoF values) yields better results than raw behavioral measurements. To support this claim, the authors should train the classifier on the underlying measures (accuracy and RT) and show reduced predictive power.

5. In the RT analysis (Fig 4), the authors show very strong correlations between the measured and predicted RT. However, it's important to disentangle the contribution of the construct of interest and that of no interest, t0. If I understand correctly, t0 is fitted for each participant, but is not directly related to memory decline in any way. Shouldn't the comparison between the measured and predicted RT values after accounting for t0 (and possibly F as well)? Furthermore - while not at the focus of this work - if F, given the collected data, F could be related to executive function, which was found to be lowered in some of the MCI participants.

6. "The number of items studies during each session varied... depending on their response times and error rates". Is this a confounding factor? My understanding is that it is not, but it would be useful to clarify that the number of items does not actively affect the inferred SoF during the experiment.

7. "The mean SoF values were identified by using the last SoF of each item..." - Could you please show a visualization of the inferred SoF values time course? It would be useful to show that the values indeed converge and stabilize. In addition, have the authors considered the sensitivity of other measures like the SD of the SoF values? This goes back into what we think the underlying source of change in MCI is. Is it an overall increase if SoF? If so, the mean (or median) is a good measure. However, if it is in fact an increase in the frequency of more rare forgetting events, than the SD or IQR might be more sensitive measures.

8. When presenting the stability and reliability of the model-based assessment scores, it would be very helpful to present the stability and reliability of the underlying behavioral measures (accuracy and mean RT). In addition, because we know that there is a difference between groups, such a difference could be driving the high correlation value of Pearson r=0.7. This is not necessarily a bad thing, because it is in fact this difference between MCI and controls that the authors are trying to capture. Still, it would be useful to report the reliability using Spearman correlation, which is less sensitive to extreme values. I would even consider reporting them separately for the control and MCI groups (see point 11 below).

9. Fig 7A: Some lessons are more discriminative than others between MCI and controls. Can the authors say something about what makes lesson more discriminative? For example, flags, art and constellations are less discriminative. Is that because they are more visual that other lessons? Because there are other strategies people might use to complete these lessons? Understanding this might get even better predictive results.

10. Fig 7B: As is often the case in such tests, there is still a lot of overlap between the distributions of the two populations. Could the authors please explicitly address this? Is it possible that in the future, a more sensitive measure for predicting the onset of MCI be the *change* in SGMA over sessions? I think this is exactly the point that the authors are trying to make. If this is the case, the reporting the reliability (using correlations of ICC values) in each group separately, and particularly in the control group, is of great importance.

11. The analysis of the diagnostic accuracy of SGMA is a very strong part of the paper, and a great contribution to the field.

12. I think that this analysis should be complemented with an similar analysis that is based on the measured accuracy and RT measures, as the underlying building blocks of SGMA. It might be the case that they are as predictive as SGMA (which is perfectly fine), but knowing it would be very helpful. Please correct me if I'm wrong, because it's possible that the adaptive nature of this task actually makes accuracy and RT less sensitive (as the platform pushed participants toward a common accuracy value, for example, while inferring the SoF. If this is the case, I think that the readers would greatly benefit from a clear explanation of this point.

13. The authors write that typical assessments that are based on test accuracy confound memory loss and deficits in accessing a memory (e.g., due to executive dysfunction), but that SGMA is potentially immune to this confound. Could you please elaborate on why SGMA is potentially immune to this confound? Furthermore, given that there are patients that exhibited executive dysfunction in this study's cohort, does anything in their SGMA scores or otherwise reflect this dysfunction?

14. Given the strong Sex effect, wouldn't it make sense to run the classification analysis with both SGMA and Sex as inputs in a multiple-logistic classifier? Perhaps this is what the authors have already done. Similarly, did the authors consider using both recognition SGMA and recall SGMA in a single prediction model?

Minor comments

1. Intro: The authors write "... as they lacked reliability when used in isolation". Please explain what this means. Is that test-retest reliability? Was the detection accuracy of 90% not reliable?

2. Intro: The authors write: "The present study demonstrates, for the first time, the viability of this approach". Is that really the first time researchers use computational phenotyping to differentiate healthy individuals from diseased ones? Please soften this claim, or make it specific to the test case in question (detection of MCI).

3. Intro: There is a typo in "an individual can hold on to *be* a newly..."

4. Intro: "individuals with MCI would exhibit higher SGMA scores" - should be "lower", right?

5. In "Adaptive Memory Assessment", the reference "Fig 2" should actually be to "Fig 3".

6. Currently, Fig 5 is a bit hard to read. It would make more sense to present the 1st to 9th deciles in this plot, and present the full plots (including the 10th decile) in the supplementary.

7. Fig 6: "from panel B" should probably be "from panel A".

8. The longitudinal aspect of this study, with so many repeated measures per participant, is very impressive. The authors have used this to show explicitly that there is minor improvement due to practice (having a small practice effect is an advantage in terms of test-retest reliability), and that this effect is attenuated in MCI. Given the SGMA (like any other single test) still shows a large fraction of false-positive "diagnosis" of MCI in the logistic classifier, it would have been very useful to show the ability of identifying a conversion from healthy control to MCI. This is obviously not at the hands of the authors, however, if they are able to track these participants further into the future, and show that SGMA is able to identify MCI onset, this would be an extremely strong result.

Reviewer #2: The authors report results from a mobile cognitive test of memory consolidation and forgetting performed at home on a weekly basis through a year in patients with mild cognitive impairment (MCI) and a control group of healthy age-matched individuals. The model compared test results to group members.

The method description, data processing and statistics are sound and descibed well.

The introduction and discussion are focused and well balanced.

Overall, a significant contribution to the field.

Reviewer #3: The authors developed the Seattle-Groningen Memory Assessment to address some constraints of conventional neuropsychological tests; specifically their reliance on in-person testing, vulnerability to practice effects, and lack of adaptive features. They conducted a longitudinal study lasting up to one year, recruiting 27 healthy controls and 24 people with amnestic MCI. On a weekly basis, participants engaged in 8-minute memory tasks delivered online.

By applying a mathematical model of memory consolidation to trial-by-trial metrics (both response time and accuracy), the team established a "Speed of Forgetting" (SoF) parameter, which was then normalised as the SGMA score. The findings indicated that the SGMA scores were reliable (r = 0.71) and reached a diagnostic accuracy of 87% differentiating between controls and MCI, which is equivalent to the clinician-led MoCA. T tool proved to be stable across various modalities (recall vs recognition) and different sets of study materials.

It’s a rigorous study addressing a genuine issue in clinical practice and with the adjustments below I think it’d be a valuable contribution to the literature.

Major Points

- My biggest query concerns the tool’s current ability to do the “continuous monitoring” aspect mentioned in the title. While the work successfully differentiated groups (i.e. cross-sectional utility), it’s not yet shown that the tool can track an individual’s specific trajectory of cognitive decline. The abstract asserts that the score is "only barely affected by practice effects". But Figure 7C and Table 1 indicate a significant positive fixed effect for "Week" (p<0.001). Although the weekly increase is small (0.17 points), it would accumulate to a ~8.8 point gain over a full year. While it’s encouraging that the increase is smaller in people with MCI (i.e. there’s a separation of cases vs controls), I worry that this persistent upward slope creates a confound for a tool designed to track cognitive decline. Describing the tool as less affected than traditional tests would be more precise than claiming it is ‘resistant’, as it’s clearly not immune. Related to this, I'd be interested to know the mean number of repetitions per item for MCI vs control groups e.g. if MCI patients required significantly more repetitions to reach the same level of accuracy within the 8 minutes, it could demonstrate the ‘burden’ of the task perhaps more clearly than just the final SGMA score?

- I'm impressed by how high engagement seems to have been in this cohort (engaged with weekly testing for 48.5 weeks). Do the authors have any indication why this might be? Is it something unique to this cohort? Or are there lessons from your approach which you think contributed to this which others could learn from? Either way I’d be interested to hear the authors’ perspective on this.

- Related to the above, would this engagement be lower for people with greater cognitive impairment? Would this be an issue for picking up incident dementia i.e. if as people ‘convert’ from MCI to dementia, would a potential inability to engage with the tool impair the ability to detect the ‘conversion’? While the authors mention the necessity of intact sensory/motor function, I think it might also warrant some mention of the potential for psychological distress or frustration felt by patients who perceive their own cognitive decline through repeated testing.

- There isn’t contextualisation of these results between SGMA and established digital platforms Cogstate or Cognitron. SGMA’s primary differentiator seems to be its digital twin modelling of the forgetting curve, whereas other tools frequently rely on raw performance data. The discussion would benefit from a more explicit explanation of why tracking latent parameters through modelling is superior to the raw data tracking used by other systems.

- The abstract has quite a lot of use of subjective intensifiers e.g. "highly diagnostic”, "highly stable", "only barely affected" which risk overstating some of the results, especially for an n=51 pilot study

Minor Points

- The tool seems to currently focus on memory. Do the authors intend for this to be used as a standalone tool or alongside other tools testing other cognitive domains or planning to add additional modalities cognitive domains?

**********

**Figure resubmission:**

**Reproducibility:**

To enhance the reproducibility of your results, we recommend that authors of applicable studies deposit laboratory protocols in protocols.io, where a protocol can be assigned its own identifier (DOI) such that it can be cited independently in the future. Additionally, PLOS ONE offers an option to publish peer-reviewed clinical study protocols. Read more information on sharing protocols at https://plos.org/protocols?utm_medium=editorial-email&utm_source=authorletters&utm_campaign=protocols

# Author comment: Author response to Decision Letter 1

**DOI:** 10.1371/journal.pdig.0001686.r002

21 Jul 2026

Attachment Submitted filename: Blaarkop_PLOSDH_R2R.docx

# Editor report: Decision Letter 1

**DOI:** 10.1371/journal.pdig.0001686.r003

**Authors:** Pengxu Wei, Peter H Charlton

10 Aug 2026

Reliable Detection and Continuous Monitoring of Memory Dysfunction in Mild Cognitive Impairment and Healthy Aging Through Adaptive Computational Phenotyping

PDIG-D-26-00101R1

Dear Dr. Stocco,

We are pleased to inform you that your manuscript 'Reliable Detection and Continuous Monitoring of Memory Dysfunction in Mild Cognitive Impairment and Healthy Aging Through Adaptive Computational Phenotyping' has been provisionally accepted for publication in PLOS Digital Health.

Before your manuscript can be formally accepted you will need to complete some formatting changes, which you will receive in a follow-up email from a member of our team.

Please note that your manuscript will not be scheduled for publication until you have made the required changes, so a swift response is appreciated.

IMPORTANT: The editorial review process is now complete. PLOS will only permit corrections to spelling, formatting or significant scientific errors from this point onwards. Requests for major changes, or any which affect the scientific understanding of your work, will cause delays to the publication date of your manuscript.

If your institution or institutions have a press office, please notify them about your upcoming paper to help maximize its impact. If they'll be preparing press materials, please inform our press team as soon as possible -- no later than 48 hours after receiving the formal acceptance. Your manuscript will remain under strict press embargo until 2 pm Eastern Time on the date of publication. For more information, please contact digitalhealth@plos.org.

Thank you again for supporting Open Access publishing; we are looking forward to publishing your work in PLOS Digital Health.

Best regards,

Pengxu Wei

Academic Editor

PLOS Digital Health

***********************************************************

**Additional Editor Comments (if provided):**

**Reviewer Comments (if any, and for reference):**
